# The Role of Methylation in Chronic Lymphocytic Leukemia and Its Prognostic and Therapeutic Impacts in the Disease: A Systematic Review

**DOI:** 10.1155/2024/1370364

**Published:** 2024-02-23

**Authors:** Sevastianos Chatzidavid, Christina-Nefeli Kontandreopoulou, Nefeli Giannakopoulou, Panagiotis T. Diamantopoulos, Christos Stafylidis, Marie-Christine Kyrtsonis, Maria Dimou, Panayiotis Panayiotidis, Nora-Athina Viniou

**Affiliations:** ^1^Hematology Unit, First Department of Internal Medicine, Laikon General Hospital, National and Kapodistrian University of Athens, Athens, Greece; ^2^Thalassemia and Sickle Cell Disease Center, Laikon General Hospital, Athens, Greece; ^3^Second Department of Hematology, Iaso General Hospital, Athens, Greece; ^4^Hematology Section of the First Department of Propaedeutic Internal Medicine, Laikon University Hospital, Athens, Greece; ^5^Department of Hematology and Bone Marrow Transplantation Unit, National and Kapodistrian University of Athens, School of Medicine, Laikon General Hospital, Athens, Greece; ^6^Hematology Department, Iatriko Kentro Palaiou Falirou, Athens, Greece

## Abstract

Epigenetic regulation has been thoroughly investigated in recent years and has emerged as an important aspect of chronic lymphocytic leukemia (CLL) biology. Characteristic aberrant features such as methylation patterns and global DNA hypomethylation were the early findings of the research during the last decades. The investigation in this field led to the identification of a large number of genes where methylation features correlated with important clinical and laboratory parameters. Gene-specific analyses investigated methylation in the gene body enhancer regions as well as promoter regions. The findings included genes and proteins involved in key pathways that play central roles in the pathophysiology of the disease. Τhe application of these findings beyond the theoretical understanding can not only lead to the creation of prognostic and predictive models and scores but also to the design of novel therapeutic agents. The following is a review focusing on the present knowledge about single gene/gene promoter methylation or mRNA expression in CLL cases as well as records of older data that have been published in past papers.

## 1. Introduction

Chronic lymphocytic leukemia (CLL) is characterized by the accumulation of clonal B cells in peripheral blood, bone marrow, and secondary lymphoid organs. It is the most common leukemia in adults of Western countries and accounts for approximately 2–35 percent of all leukemias in the United States [1]. CLL is more common in men with a male to female ratio ranging from 1.2 : 1 to 1.8 : 1 [1, 2] and is considered to be a disease of the elderly as the median age at diagnosis is 70 years and an incidence increasing rapidly with increasing age [3]. Despite characterized by a common morphology and immunophenotype pattern, CLL shows a variable clinical behavior spectrum. At one end, some CLL patients have a very indolent disease course and may not require treatment for many years, while at the other end, some patients present a very aggressive disease early from diagnosis and require prompt treatment with lower survival rates despite therapy [4].

In terms of genetic pathobiology, at least one of the four common chromosomal abnormalities (deletion 13q14, trisomy 12, deletion 11q22-23, and deletion 17p12) can be detected by interphase fluorescence in situ hybridization in most patients [5]. Most CLL tumors have myriads of somatic gene mutations. Of these, tumor protein 53 (TP53), ataxia telangiectasia mutated (ATM), neurogenic locus, notch homolog protein (NOTCH), and subunit 1 of the splicing factor 3b protein complex (SF3B1) genes are the most frequently mutated at diagnosis [6, 7]. Several other gene mutations seen in CLL tumors are involved in important cellular signaling pathways. NOTCH signaling, B-cell receptor (BCR) signaling, Toll-like receptor, mitogen-activated protein kinase/extracellular signal-regulated kinase (MAPK-Erk) pathway, nuclear factor kappa-light-chain enhancer of activated B cells (NF-kB) signaling, chromatin modifiers signaling, cell cycle signaling, DNA damage signaling, and RNA splicing are some of the many pathways investigated [6–10]. Moreover, it is well documented that the interaction between CLL tumor cells and antigens is regulated by the somatic hypermutation load of the immunoglobulin heavy chain variable region genes [11].

In the complex domain of the tumor microenvironment, CLL cells seem to influence the kind and arrangement of the cells surrounding them, and they are highly dependent on signals from these cells for their own survival and proliferation, encouraging a leukemia-supportive and immunosuppressive microenvironment [11].

Methylation of DNA is an enzyme-mediated modification of DNA structure without interfering in the specific sequence of the base pairs for the gene encoded. Although DNA methylation is important in normal biologic processes, aberrant patterns of methylation are observed in several malignancies. Specifically, two patterns have been mainly described, namely, large sites of global hypomethylation along the genome and localized areas of hypermethylation at CpG islands, within the gene promoter regions. Ιn more and more studies in neoplastic diseases in recent years, there has been an emergence of evidence that methylation of the promoter regions of hundreds of genes, including tumor suppressor genes, results in the failure to express their normal purpose. In other cases, DNA methylation may represent an early step in the pathway by which normal tissue cells undergo tumorigenesis. Some other possible mechanisms for mutational induction secondary to DNA methylation include failure to produce DNA repair proteins that normally protect from mutations and predisposition to increased oxidative DNA damage, resulting in increased mutation potential [12].

Abnormal DNA methylation has been documented in many solid neoplasms, including breast cancer, lung, prostate, colorectal cancer, and melanoma. As described above, hypermethylation of promoter regions inhibits the expression of tumor suppressor genes while hypomethylation activates an oncogene expression. Global hypomethylation is also of crucial role, leading to genomic and chromosomal instability. For instance, BRCA1/2 genes are two of the most studied genes in breast cancer. The hypermethylation of their promoter regions is reported to result in their inactivation and consequent increased risk of breast cancer [13].

As regards CLL, a global DNA methylation pattern was reported to be relatively stable during the disease course and similar both in resting and proliferative cell compartments, implying that aberrant methylation may present as an early leukemogenic event with early indications of specific gene epigenetic changes in CLL samples being described over 30 years ago [14]. Global DNA hypomethylation was reported few years later [15].

Except CLL, abnormal function of basic methylation‐related enzymes is widely reported in several hematological malignancies, such as in myelodysplastic syndromes, myeloproliferative neoplasms, acute myeloid leukemia, T-cell acute lymphoblastic leukemia, and diffuse large B‐cell lymphoma. The final DNA methylation patterns are suggested to be frequently lineage specific and accompanied by specific secondary mutations [16].

The research in this field led to the study and discovery of a large number of genes whose methylation plays an important role in their regulation, while certain correlations with clinical and laboratory parameters were also identified. The streptothricin acetyltransferase, alpha (SAT-*α*) gene, whose methylation levels were found to be an independent marker of poor treatment-free survival was an early example [17].

With the appearance and application of newer technics such as whole-genome bisulfite sequencing and DNA methylation arrays, global DNA hypomethylation was confirmed and it was reported that gene body and enhancer regions rather than promoter regions were primarily involved [18, 19] whereas hypermethylation was described to occur mainly in transcribed genomic regions [20] and regions affecting other regulatory mechanisms such as spliceosome [21]. After the CLL genome was discovered to be globally hypomethylated, research for aberrantly methylated oncogene targets revealed that hypomethylation of the B cell lymphoma 2 (BCL2) gene, an important antiapoptotic gene, correlated with higher protein expression in CLL [22]. In the following studies, multiple drug-resistance protein 1 (MDR1) and T-cell leukemia/lymphoma 1 (TCL1) genes were reported to be both hypomethylated and upregulated in CLL [23, 24]. Moreover, the nuclear factor of activated T-cell 1 (NFATc1) gene hypomethylation was also identified and was further shown to be associated with increased mRNA and protein expression, suggesting hypomethylation as a mechanism of constitutive activation of NFATc1 expression in CLL. Through subsequent findings, however, it was discovered that the activation of oncogenes through DNA hypomethylation was a rather infrequent lesion in CLL [25].

To date, several studies have reported gene promoter hypermethylation in CLL patients using both global and single gene approaches. The affected genes include tumor suppressors, transcription factors, genes involved in survival and proliferation, genes with prognostic impact, and microRNA genes. There are several methods that have been used to study gene methylation in chronic lymphocytic leukemia. Each approach has its own advantages and limitations, and the choice of the method depends on the specific research question being addressed. Bisulfite sequencing is considered the gold standard method for analyzing DNA methylation at single-nucleotide resolution. Bisulfite treatment of DNA converts unmethylated cytosine to uracil, while leaving methylated cytosine unchanged. After polymerisation chain reaction (PCR) amplification and sequencing, the pattern of cytosine-to-thymine conversion can be used to determine the methylation status of individual CpG sites. Methylation-specific PCR is a PCR-based method that uses primers specific for either methylated or unmethylated DNA to amplify a region of interest. By comparing the intensity of PCR products amplified using methylated-specific versus unmethylated-specific primers, the methylation status of a given region can be determined. Infinium methylation arrays are microarray-based platforms that can interrogate the DNA methylation status of thousands of CpG sites across the genome. Infinium arrays rely on bisulfite conversion of DNA, followed by hybridization with probes that distinguish between methylated and unmethylated DNA at each CpG site. Methylation-sensitive restriction enzyme digestion relies on the use of restriction enzymes that are sensitive to DNA methylation. By digesting genomic DNA with these enzymes and comparing the resulting fragment patterns with and without prior treatment with a DNA methyltransferase, the methylation status of specific regions can be inferred.

## 2. The CLL Methylome from a Gene-Specific Perspective

Early in 2004, using genome-wide screening for aberrant promoter methylation in CLL samples, 193 sequences were identified as novel targets for aberrant methylation in CLL. Among them, 173 were homologous to specific genes such as glutamate metabotropic receptor 7 (GRM7), cell division protein kinase 6 (CDK6), T-box transcription factor 3 (TBX3), paired box 5 (PAX5), and protein tyrosine phosphatase nonreceptor type 1 (PTPN1) genes [19]. In another study, where DNA methylation was compared within immunoglobulin heavy-chain gene variable region (IGVH) mutated/unmutated subgroups of CLL, it was reported that in IGVH-unmutated cells, tumor suppressor genes such as von Hippel–Lindau (VHL), Abelson interactor 3 (ABI3), and immunoglobulin superfamily member 4 (IGSF4) were found hypermethylated while genes associated with cell proliferation and migration such as adenosine A3 receptor (ADORA3) and perforin 1 (PRF1) were hypomethylated [26].

Regarding microRNAs, a genome-wide profiling in CLL patients led to the detection of 128 microRNAs carrying aberrantly methylated promoters. Hypermethylated loci included the promoter regions of miR-9-2, miR-124-2, miR-129-2, miR-551b, and miR-708, while miR-21, miR-29a/b-1, miR-34a, miR-155, miR-574, and miR-1204 gene promoters were found hypomethylated [27].

When DNA methylation was studied in paired diagnostic and follow-up samples from IGVH mutated and unmutated CLL patients, genes with prognostic significance including chronic lymphocytic leukemia upregulated 1 (CLLU1), lipoprotein lipase (LPL), zeta chain of T-cell receptor-associated protein kinase 70 (ZAP70) and NOTCH1, the epigenetic regulators histone deacetylases 9 and 4 (HDAC9/4), and DNA methyltransferase 3, beta (DNMT3B), were reported to be aberrantly methylated [14]. Another study used the methylation levels of five specific CpGs to track the cellular origin of CLL, where they seemed to act as enhancers. The varying methylation levels of these biomarkers did not translate into gene expression changes, suggesting that they may not have a functional impact but represented a stable molecular mark [28].

In the context of normal B-cell maturation, CLL DNA methylation was reported to be highly enriched in enhancer and promoter regions, especially in regions of transcriptional elongation and in genes involved in B cell- and lymphocyte-related processes and pathways. Regions targeted for hypomethylation during B cell maturation showed highly significant enrichment for the following six transcription factor families: activator protein 1 (AP-1), early B-cell factor (EBF), runt-related transcription factor (RUNX), octamer-binding transcription factor (OCT), interferon regulatory factor (IRF), and NF-kB [20].

In future, it will be crucial to further develop more efficient and accurate prognostic tools that incorporate clinical, cytogenetic, and molecular data. Therapeutic strategies and agent design are constantly reformed in order to pursue discoveries about the biology of the disease, the pathways involved, and the mechanisms of drug resistance.

**Figure 1:**
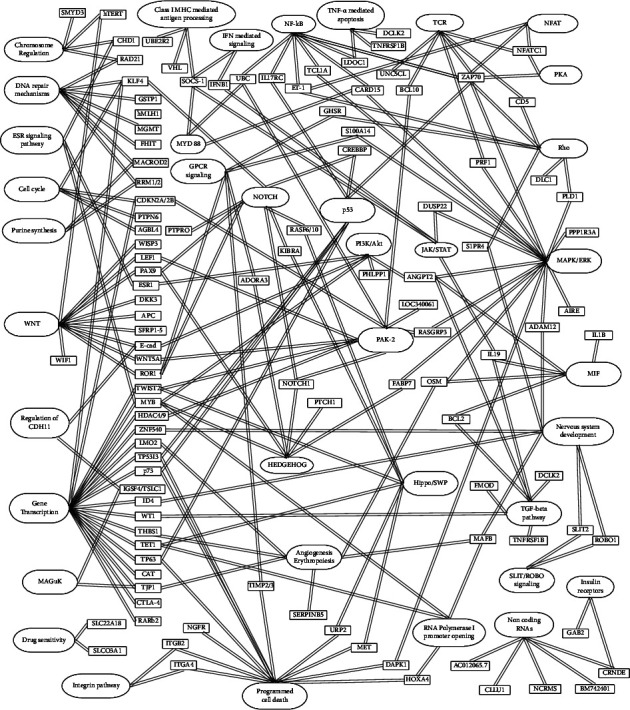
Schematic representation of the interaction between genes and major biological pathways (created with GitMind®: https://gitmind.com/).

To capture the latest discoveries, this review focuses on the present knowledge about single gene/gene promoter methylation in CLL biology while also recording older data that have been published in past papers. Through a review of 126 articles indexed in PubMed, we ended up recording data for 133 genes. Then, we classified them in the following tables according to their associated biological pathways and the related data on CLL studies. It is reasonable for several genes to be associated with more than one pathway. Α characteristic attempt to capture the correlations between most genes and pathways is presented in Figure 1.

In the following tables are presented the genes we identified in our search, with information on their function, as well as the findings of studies regarding CLL. In each table, we introduced in detail, data on the study of methylation or mRNA expression of single genes in CLL patients that showed interesting correlations with clinical and laboratory data.

### 2.1. Chromosome Maintenance and the Cell Cycle Process

#### 2.1.1. SE Translocation and MYN Domain Containing 3 (SMYD3) and Human Telomerase Reverse Transcriptase (hTERT) Genes (Table 1)

SMYD3 is a chromatin modifier that is involved in the development and progression of several malignancies. Methylation levels in specific SMYD3 gene promoter CpG sites were reported to independently predict time to treatment [29].

Hypermethylation of the hTERT gene in CLL was one of the first to be described. TERT acts as a subunit of telomerase, preventing chromosomal degradation after DNA replication. It was reported that hTERT promoter hypermethylation led to decreased telomerase activity and was associated with superior overall survival [30].

### 2.2. DNA Repair Mechanisms and Purine Biosynthesis

#### 2.2.1. Ribonucleotide Reductase Subunits 1 and 2 (RRM1 and RRM2) Genes (Table 2)

Ribonucleotide reductase is required for DNA replication and repair and consists of RRM1 and RRM2 proteins. In CLL patients, RRM1 mRNA expression was higher in patients without anemia, absence of lymphadenopathy, and 17p gene deletion. Moreover, abnormal lactate dehydrogenase (LDH) levels and higher Rai stage were associated with lower RRM1 mRNA levels. Higher expression of RRM2 mRNA was detected in patients without lymphadenopathy, Rai stage 0, and trisomy 12. The methylated status of RRM1 promoter significantly correlated with lymphadenopathy presence. The methylated status of RRM1 promoter correlated also with about 4 times lower levels of RRM1 mRNA and with about 10 times lower levels of RRM2 mRNA [39].

### 2.3. Gene Transcription

#### 2.3.1. Cytotoxic T-Lymphocyte-Associated Protein 4 (CTLA-4) Gene (Table 3)

CTLA-4 protein is involved in gene transcription and acts as an immune checkpoint to regulate T-cell function. Mutations in its gene have been associated with insulin-dependent diabetes mellitus, Grave's disease, Hashimoto thyroiditis, celiac disease, systemic lupus erythematosus, and other autoimmune diseases. In CLL cases, CTLA4 gene was hypomethylated in the first exon region and body region. Compared to healthy controls, CTLA4 gene had a 128-fold higher expression in CLL samples [37, 59].

### 2.4. RNA Polymerase I Promoter Opening

#### 2.4.1. V-Maf Musculoaponeurotic Fibrosarcoma Oncogene Homolog B (MAFB) Gene (Table 4)

MAFB gene has been associated with survival parameters in CLL cohorts where differentially hypermethylated regions correlated with inferior post-treatment survival. The protein encoded by MAFB gene is a basic leucine zipper (bZIP) transcription factor with an important role in the regulation of hematopoiesis and nervous system development [66].

### 2.5. Programmed Cell Death/Cell Apoptosis and p53 Signaling

#### 2.5.1. Integrin Subunit Alpha 4 (ITGA4) Gene (Table 5)

ITGA4 is capable of cell adhesion molecules and fibronectin binding and is involved in apoptotic and integrin pathways. It is reported to be deregulated in CLL with adverse clinical features, suggested as a negative prognostic factor related to a more aggressive course and shorter time to treatment. Protein expression is regulated at the mRNA level and in a methylation-regulated manner. Hypermethylation at specific ITGA4 CpG sites was a common phenomenon in the del13q14+ samples. Moreover, it was demonstrated that the methylation status of the ITGA4 gene at CpG site 1 may have a prognostic role [70].

### 2.6. Transforming Growth Factor-*β* (TGF-*β*) Pathway

#### 2.6.1. Doublecortin-Like Kinase 2 (DCLK2) and Tumor Necrosis Factor Receptor Superfamily Member 1B (TNFRSF1B) Genes (Table 6)

Retrotransposons (also referred as class I transposable elements or transposons through RNA intermediates) are a type of genetic component that copy and paste themselves into different genomic locations (transposons) by retroconverting RNA into DNA via the reverse transcription process using RNA transposition intermediate. In solid neoplasms, universal hypomethylation of these elements has been described. In CLL, locus-specific hypomethylation was detected with differential expression of proximal genes, including DCLK2 and TNFRSF1B genes. Moreover, higher levels of DCLK2 and TNFRSF1B expression were associated with inferior survival. DCLK2 protein is characterized by transferase and protein tyrosine kinase activity, whereas TNFRSF1B protein by ubiquitin protein ligase binding and tumor necrosis factor (TNF) receptor activity. Both proteins are involved in TGF-*β* and TNF pathways [88].

### 2.7. Wingless-Related Integration Site (WNT) and Hedgehog Pathways

#### 2.7.1. WNT Family Member 5A (WNT5A) Gene (Table 7)

WNT5A gene encodes a receptor tyrosine kinase-like orphan receptor (ROR1) ligand with DNA-binding transcription factor activity and is involved in the regulation of p21 activated kinase 2 (PAK-2), WNT signaling, and developmental pathways during embryogenesis. It is known that its expression differs in CLL patients with worse prognosis in the IGVH-mutated subgroup. Methylation levels of all CpG sites in the WNT5A gene promoter were lower in the group of the intermediate genome methylation profile. In the memory-like and intermediate genome methylation profile groups, promoter methylation and subsequent undetectable WNT5A expression correlated with longer treatment-free survival [95].

### 2.8. NFAT and T-Cell Receptor (TCR) Signaling

#### 2.8.1. NFATC1 Gene (Table 8)

NFATC1 is part of the NFAT transcription complex. It is primarily involved in gene transcription during the immune response and acts as a downstream regulator of the BCR signaling pathway. As with many other proteins, alternative splicing leads to multiple transcription variants, which in turn may induce the expression of different cytokine genes. Moreover, NFATC1 protein is a central target for immunosuppressive agents. Regarding CLL, when the genome-wide DNA methylation study was performed, NFATC1 gene was reported to be hypomethylated and upregulated. Moreover, NFATC1 gene promoter DNA hypomethylation correlated inversely with RNA levels and was associated with Binet disease staging and thymidine kinase levels, suggesting a potential central role of NFATC1 in CLL pathobiology [105].

### 2.9. NF-kB Pathway

#### 2.9.1. Leucine Zipper Was Downregulated in Cancer 1 (LDOC1) Gene (Table 9)

LDOC1 gene encodes a leucine zipper protein and is suggested to regulate the NF-*κ*B pathway through the plasma membrane ATPase or TNF-mediated pathway of apoptosis. Its expression has been studied in oral squamous cell carcinoma and pancreatic cancer cells. Regarding CLL, unmethylated status was found in IGVH-unmutated cases [26, 111, 112].

### 2.10. MAPK/Erk Pathway

#### 2.10.1. Angiopoietin 2 (ANGPT2) Gene (Table 10)

ANGPT2 is a glycoprotein involved in angiogenesis. Conflicting data regarding the involvement of Erkand phosphoinositide 3-kinase/protein kinase B (PI3K/Akt) pathways in the regulation of ANGPT2 suggest a possible cell-type specific control for this gene in solid tumors. As regards CLL, higher levels of ANGPT2 methylation in the IGHV-mutated population showed a possible epigenetic regulation of this gene. Furthermore, it was reported that ANGPT2 expression is highly dependent on the DNA methylation status, where a lower degree of methylation was associated with a particularly poor prognosis in CLL. Importantly, the percentages of methylation showed negative correlations with ANGPT2 mRNA expression, suggesting that methylation of the ANGPT2 promoter leads to this gene silencing. CLL cases with highly methylated gene status had a more favorable prognosis. Considering that normal B cells showed high levels of ANGPT2 methylation, the apparently aberrant low ANGPT2 methylation levels found in aggressive CLL patients indicate that these latter cases lose normal epigenetic control [14, 119–121].

### 2.11. NOTCH Signaling

#### 2.11.1. CREBBP (cAMP Response Element-Binding Protein Binding Protein) Gene (Table 11)

In a small Korean cohort, genome-wide methylation profiling identified the CREBBP gene with no known relevance to CLL to be differentially methylated among other genes previously known to be affected in CLL. CREBBP gene encodes chromatin-modifying enzymes and has been described in diffuse large B cell lymphoma, acute lymphoblastic leukemia, and lung cancer [130].

### 2.12. JAK-STAT Pathway

#### 2.12.1. SOCS-1 (Suppressor of Cytokine Signaling 1) Gene (Table 12)

SOCS-1 is an inhibitor of cytokine signal transduction. Its gene expression can be positively affected by interleukins 2 and 3, erythropoietin, granulocyte macrophage colony-stimulating factor, and interferon-gamma. Gene polymorphisms of SOCS1 and its expression have been studied in several malignancies including diffuse large B-cell lymphoma and acute lymphoblastic leukemia, where, particularly, the expression level of SOCS1 was lower compared to the control group. In CLL, the SOCS1 gene body was hypermethylated in no case [135].

### 2.13. Phosphatidylinositol-3 Kinase/Protein Kinase B (PI3K/Akt) Signaling

#### 2.13.1. T-Cell Leukemia/Lymphoma 1 Oncogene (TCL1A) Gene (Table 13)

Abnormal expression of TCL1A gene in mouse B-cells led to a leukemia phenotype similar to aggressive human CLL. It is demonstrated that TCL1A physically interacts with DNA methylthansferases 3A and 3B. TCL1A is suggested to act as a coactivator of Akt, activator protein 1 (AP1), and NF-*κ*B pathways with a potential involvement in CLL cells resistance to apoptotic mechanisms. TCL1A expression seems to be strongly associated with the expression levels of ataxia-telangiesctasia mutated (ATM) gene in malignant and nonmalignant B cells. TCL1A gene promoter was hypomethylated in CLL cells and significantly correlated with TCL1A transcription enhancement [139].

### 2.14. Rho GTPases/Rhodopsin-Like Receptors

#### 2.14.1. Endothelin-1 (ET-1) Gene (Table 14)

This gene is responsible for the creation of a peptide that belongs to the endothelin/sarafotoxin family. This peptide acts as a potent vasoconstrictor and its receptors have been well studied as therapeutic targets in the treatment of pulmonary arterial hypertension. Regarding ET-1 gene expression, alternative splicing leads to several transcript variants and abnormal expression is thought to promote tumorigenic activity, with ET-1 expression being under the constant control of the NF-kB signaling pathway. In CLL, ET-1 is reported to be involved in survival, drug resistance, and growth signaling of leukemic cells. Moreover, it is reported that basal expression levels of ET-1 are affected when high methylation in the region of ET-1 gene first intron is detected [141].

### 2.15. Class I Major Histocompatibility Complex (MHC)-Mediated Antigen Processing

#### 2.15.1. Ubiquitin Conjugating Enzyme E2 R2 (UBE2R2) Gene (Table 15)

UBE2R2 is involved in Class I MHC-mediated pathways as well as in the metabolism of certain proteins. To develop a tool useful in stratifying CLL patients on a specific methylation signature basis and focusing on time to treatment, it was demonstrated that UBE2R gene methylation levels independently predicted time to treatment [29].

### 2.16. Estrogen Receptor (ESR)-Mediated Signaling

#### 2.16.1. Estrogen Receptor 1 (ESR1) Gene (Table 16)

ESR1 gene encodes a ligand-dependent transcription factor. ESR1 acts through direct binding to specific estrogen response elements and is associated with other transcription factors. ESR-mediated signaling has been studied in several solid tumors and hematological malignancies. When studied in CLL samples, ESR1 gene was amplified in 15% of the samples examined with a copy number loss frequency ranging between 1 and 10%. The ESR1 gene promoter region was methylated in one out of ten CLL samples controlled, and in normal B cell, the gene promoter was completely unmethylated [51].

### 2.17. Angiogenesis/Erythropoiesis

#### 2.17.1. Thrombospondin-1 (THBS1) Gene (Table 17)

THBS1 acts as an adhesion-specific glycoprotein. THBS1 is able to bind fibrinogen, fibronectin, laminin, and type V collagen and is involved in platelet aggregation, angiogenesis, and tumorigenesis. In CLL studies, THBS1 gene promoter methylation was detected in 50% of the samples, while in normal samples, the gene promoters were unmethylated [51].

### 2.18. Slit Glycoprotein/Roundabout Receptor (SLIT/ROBO) Signaling and Nervous System Development

#### 2.18.1. Protein Tyrosine Phosphatase Type O (PTPRO) Gene (Table 18)

The methylation levels of the PTPRO gene have been studied in the pathobiology of several neoplasms including hepatocellular cancer and various lung tumors, with indications of potential tumor suppressor features. Regarding CLL, PTPRO gene promoter was also methylated and gene expression was suppressed compared to normal B cells, with the overall expression of PTPRO being lower in CLL lymphocytes than in normal samples [51].

### 2.19. Drug Uptake and Sensitivity Mechanisms

#### 2.19.1. Solute Carrier Family 22 Member 18 (SLCO3A1) Gene (Table 19)

Methylation of the SLCO3A1 gene has been associated with survival features in CLL cohorts where specific differentially hypermethylated regions were linked to inferior post-treatment survival. SLCO3A1 gene encodes an anion transporter that is suggested to be involved in transport of inorganic cations/anions and amino acids/oligopeptides, transport of vitamins, nucleosides, and related molecules, as well as drug uptake mechanisms [66].

### 2.20. Various Other Genes Investigated

#### 2.20.1. GATA-Binding Protein 5 (GATA5) Gene (Table 20)

GATA5 contains two GATA-type zinc fingers and is related to hepatocyte nuclear factor-1alpha. Promoter methylation of the GATA5 gene has been involved in gastric cancer biology. When studied in CLL, GATA 5 gene hypermethylation was detected in 35.1% of the cases. Another study reported that genomic regions which became hypomethylated prior to specific CLL treatment initiation but also after disease relapse were enriched for binding sites of several transcription factors related to CLL pathogenesis, including GATA5. Compared to normal B-cells, these hypomethylated regions were also enriched for the GATA 5 transcription factor [51, 167].

#### 2.20.2. SH3 and Multiple Ankyrin Repeat Domain 1 (SHANK1) Genes (Table 20)

SHANK1 gene encodes a protein acting as a scaffold molecule required for the development and function of neuronal synapses. In CLL samples, a specific CpG region of the SHANK1 gene body (hg19) was reported to be hypermethylated when compared to control samples. Moreover, without yet being strongly supported, it was reported that methylation in the same CpG was detectable in blood samples collected years before CLL diagnosis [170].

#### 2.20.3. RalA-Binding Protein-Associated Eps Domain Containing 1 (REPS1), Interleukin 1B (IL1B), and ATPase Phospholipid Transporting 9B (ATP9B) Genes (Tables 21 and 22)

Specific CpG sites at which the methylation levels independently predicted time to treatment were detected and some of them located in the gene bodies of REPS1, IL1B, and ATP9B. REPS1 protein has calcium ion and SH3 domain-binding activity with involvement in vesicle transport and endocytosis. IL1B protein has interleukin-1 receptor-binding activity and is involved in MIF-mediated glucocorticoid regulation. ATP9B protein is capable of nucleotide binding and ATPase-coupled monoatomic cation transmembrane transporting and is involved in ion channel transport and cardiac conduction mechanisms [29].

### 2.21. Noncoding RNAs

#### 2.21.1. Colorectal Neoplasia Differentially Expressed (CRNDE) Gene (Table 23)

The expression of the CRNDE gene is increased in proliferating tissues and is involved in the expression of genes associated with metabolism and in neoplasms such as colorectal adenomas and adenocarcinomas. Its related transcription is inversely regulated by insulin and insulin-like growth factors. When studied in CLL cell lines, CRNDE was downregulated and the methylation level of CRNDE promoter was higher than in normal B cells. After exposure to demethylating agents, an increase of CRNDE expression levels was reported [183].

## 3. Conclusion

Some studies have focused on the potential clinical impact of DNA methylation in CLL phenotypes. Based on the findings abovementioned, time to treatment, an essential feature of CLL management, was reported to be predicted by the methylation status of SMYD3 gene promoter and UBE2R gene methylation levels [29]. Methylation levels in specific CpG sites located in the gene bodies of REPS1, IL1B, and ATP9B genes also correlated with the time to treatment [29].

Regarding overall survival, hTERT gene promoter hypermethylation was associated with superior overall survival [30], and specific hypermethylated MAFB gene loci correlated with inferior post-treatment survival [66]. Moreover, locus-specific hypomethylation of retrotransposons proximal to DCLK2 and TNFRSF1B was related with higher levels of DCLK2 and TNFRSF1B expression and subsequent inferior survival [88]. When CLL patients were classified upon specific IGVH mutation patterns, promoter methylation of the WNT5A gene showed longer treatment-free survival in a subgroup of the patients [95]. Lower levels of DNA methylation of the ANGPT2 gene were related to a particularly poor prognosis in CLL patients [14, 121].

Findings that could be useful in staging and correlating methylation with pre-existing prognostic factors in CLL include RRM1/2 expression, the promoter methylation status that correlated with lymphadenopathy, 17p gene deletion, Rai stage, and trisomy 12 [39], and the documentation of hypermethylation at specific ITGA4 CpG sites in del13q14+ samples [70]. NFATC1 gene promoter hypomethylation was associated with Binet disease staging [105].

Drug sensitivity and mechanisms of resistance development are another important area of study in CLL. Hypermethylation of specific SLCO3A1 gene loci, a gene which among others is involved in drug uptake mechanisms, has also been associated with inferior post-treatment survival [66]. Another gene, ET-1, is related with drug-resistance potential and its expression is affected upon high methylation levels in its first intron region in patients with CLL [141].

Α last finding that could theoretically have a clinical application in CLL cases is about the SHANK1 gene. A specific CpG locus of its body (hg19) was hypermethylated when compared to control samples. Also, it was even weakly supported that methylation in the same CpG was detectable in blood samples collected years before CLL diagnosis [170]. It is a finding that, if confirmed, is very interesting and could be used in panels for early diagnosis of the disease, certainly in combination with other markers in the context of a population screening program or the surveillance of high-risk patients. Certainly, such methods of investigation have their peculiarities and must be applied after appropriate research and discussion in order to be necessary or beneficial for the patients.

Our findings suggested that certain methylation patterns have been associated with disease progression and survival features and may be used as prognostic markers. In addition, some studies have investigated the use of hypomethylating agents as a part of a therapeutic strategy in CLL.

Regarding findings from previous studies that focused on studying large numbers of genes, data are generally limited but quite intriguing. Kanduri et al. applied high-resolution methylation microarrays (27 578 CpG sites) to CLL samples, which were classified in IGHV-mutated (favorable) and IGHV-unmutated/IGHV3-21 (poor-prognostic) subsets. Results demonstrated significant differences in methylation patterns between these subgroups. In IGHV-unmutated cases, they reported methylation of known or potent tumor suppressor genes (for example, VHL, ABI3, and IGSF4) as well as unmethylated genes involved in cell growth and tumor progression (ADORA3 and PRF1). In contrast, these latter genes were silenced by methylation in IGHV-mutated patients. Moreover, they reported the reinducing of four methylated tumor suppressor genes (including VHL and ABI3) in IGHV-unmutated samples using the methyl inhibitor 5-aza-2′-deoxycytidine [26]. Pei et al. reported a genome-wide DNA methylation analysis with 1,764 gene promoters being identified as differentially methylated in at least one sample when compared with normal B cell samples. Aberrant hypermethylation was discovered in all HOX gene clusters and a significant number of WNT signaling pathway genes. The NFATc1p2 promoter and first intron hypomethylated status correlated with the upregulation of both NFAT protein expressions [25]. Kulis et al. performed a wide analysis of the DNA methylome in normal B cells and CLL samples. They identified widespread hypomethylation targeting mainly the gene body and enhancer regions, suggesting that DNA methylation may be functionally relevant beyond promoter regions. Moreover, they reported that distinct patterns in DNA methylation were recorded between different CLL subtypes [18]. Cahill et al. used high-resolution 450 K arrays to analyze samples from IGHV-mutated or untreated and IGHV-unmutated or treated patients. They identified 2239 differentially methylated CpG regions between IGHV-mutated and -unmutated patients, where the majority of the regions were placed outside annotated CpG islands. Known CLL prognostic genes (i.e., LPL, ZAP70, and NOTCH1), epigenetic regulators (HDAC9/4 and DNMT3B), B-cell signaling, and numerous TGF-*β* and NF-*κ*B/TNF pathway genes were differentially methylated between the subgroups [14]. Barrow et al. reported 490 differentially methylated regions after exposure to therapy. Among them, 31 were CLL related. Seventeen genes were classified as differentially expressed, following treatment in an independent cohort. Methylation of the HOXA4, MAFB, and SLCO3A1 differentially methylated regions associated with post-treatment patient survival and HOXA4 displayed the strongest association. Reinducing of HOXA4 expression in cell lines and primary CLL cells increased apoptosis following treatment with fludarabine, ibrutinib, and idelalisib [66]. Lastly, Zhang et al. identified 34,797 differentially methylated positions related to CLL. Most of them were hypomethylated and located in gene body sites. They combined these positions with existing DNA methylation and RNA sequencing data and identified regions associated with 1,130 genes whose expression was significantly different in CLL samples [37].

The limitations of this study include possible evidence selection bias because data from statistically significant studies are more likely to be published than those that are not statistically significant. Moreover, in some limited reports, it was not stated whether the methylation study included the gene body, the promoter region, or some other related loci. Lastly, some papers did not reported correlations with patients' clinical data such as sampling time, staging, or prognostic and treatment features.

To summarize, there are still many mechanisms that need to be investigated in order to define the extent of global aberrant DNA methylation in different prognostic groups, the fundamental role of DNA methylation in sites other than CpG islands, and the interaction of DNA methylation with other regulatory processes in the pathogenesis of CLL. Moreover, with the prospect that there is and will continue to be increasing data on gene methylation, it should be noted that a large number of findings will concern passenger DNA methylation events that need to be identified accordingly.

In conclusion, at some points, the research raises even more questions than answers. Further research is still needed to fully understand the complex interplay between DNA methylation and other epigenetic and genetic alterations in CLL and to develop more effective targeted therapies and prognostic stratification tools for this disease.

## Figures and Tables

**Table 1:** Chromosome maintenance and cell cycle process.

Gene names	Related pathways	Findings in CLL	Ref.
SMYD3	Chromatin organization	Methylation levels in specific CpG sites independently predicted time to treatment	[29]
hTERT	Chromosome maintenance signaling by WNT	Promoter hypermethylation was associated with superior overall survival	[30]
CDKN2A/2B	Capable of inducing cell cycle arrest in G1 and G2 phases and regulation of activated PAK-2 gene expression (transcription)	Both genes' promoters have been found variously hypermethylated in CLL cases among different studies; common point of these studies was that hypermethylation of CDKN2A and CDKN2B was mutually exclusive in CLL cases	[31–34]
KLF4	G1-to-S transition of the cell cycle after DNA damage through p53 gene expression (transcription), NOTCH	Aberrant methylation of the KLF4 promoter was significantly associated with gene expression levels compared to normal samples after B cell activation, KLF4 expression was reported to be downregulated	[35]
PTPN6	Cell growth, differentiation, mitotic cycle, and oncogenic transformation	In advanced Rai stage cases, aberrant methylation of PTPN6 promoter reached 70% in the samples examined	[36]
AGBL4	Metabolism of proteins actin and tubulin folding	AGBL4 expression was reported to be reduced in patients with hypermethylated promoter regions and hypomethylated body regions	[37, 38]

CLL: chronic lymphocytic leukemia, Ref.: reference, SMYD3: SE translocation and MYN-domain containing 3 protein, hTERT: human telomerase reverse transcriptase, CDKN2A/2B: cyclin-dependent kinase inhibitors 2A and 2B, KLF4: Krüppel-like factor 4, PTPN6: tyrosine-protein phosphatase nonreceptor type 6, AGBL4: ATP/GTP binding protein like 4, WNT: wingless-related integration site, PAK-2: P21 activated kinase 2, NOTCH: neurogenic locus notch homolog protein.

**Table 2:** DNA repair mechanisms and purine biosynthesis.

Gene names	Related pathway	Findings in CLL	Ref.
RRM1 and RRM2	Pyrimidine deoxyribonucleotides biosynthesis and purine nucleotides de novo biosynthesis	RRM1 mRNA expression was higher in patients without anemia, absence of lymphadenopathy and 17p gene deletion, abnormal LDH and higher Rai stage were reported to be associated with lower RRM1 mRNA levels, and higher expression of RRM2 mRNA was detected in patients without lymphadenopathy, Rai stage 0, and trisomy 12	[29, 39]
RAD21	Separation of sister chromatids and cell cycle DNA repair mechanisms	There are differences in RAD21 promoter methylation proportion among patients, RAD21 inactivation via methylation may affect DNA repair mechanisms and amplify self-renewal potential of CLL cells	[40–45]
MGMT and hMLH1	DNA damage reversal homology-directed repair	MGMT promoter region was rarely identified to be hypermethylated, and hMLH1 promoter was reported to be hypermethylated in a small case series of indolent CLL with later Richter's transformation	[32, 46–50]
FHIT	Loss of its activity results in replication stress and DNA damage	In a limited series of CLL cases studied, FHIT promoter was reported to be hypermethylated	[51–53]
GSTP1	Important regulatory features in detoxification, antioxidative damage innate immune system	GSTP1 promoter hypermethylation was reported in 2.7% of the samples controlled	[51, 54–56]
MACROD2	DNA damage response purine nucleoside metabolic process	MACROD2 expression was demonstrated to be lower in cases with hypermethylated promoter regions and hypomethylated body regions	[37, 57]
ADORA3	Activation of the NF-kB pathway, purinergic signaling, GPCR signaling	Methylated gene body is reported in IGVH-mutated cases	[26, 58]

CLL: chronic lymphocytic leukemia, Ref.: reference, RRM1: ribonucleotide reductase subunit 1, RRM2: ribonucleotide reductase subunit 2, RAD21: double-strand-break repair protein rad21, MGMT: methylguanine methyltransferase, hMLH1: human mutL homolog 1, FHIT: fragile histidine triad, GSTP1: glutathione S-transferase p1 gene, MACROD2: mono-ADP ribosylhydrolase 2, ADORA3: adenosine A3 receptor, NF-kB: Nuclear factor kappa B, GPCR: G protein-coupled receptor, LDH: lactic dehydrogenase, IGVH: immunoglobulin variable heavy chain gene.

**Table 3:** Gene transcription.

Gene names	Related pathway	Findings in CLL	Ref.
CTLA4	Gene expression (transcription) CD28 costimulation	CTLA4 was hypomethylated in the first exon region and body region and had 128-fold higher expression compared to healthy controls	[37, 59]
LMO2	Angiogenesis and erythropoiesis gene expression (transcription), Assembly of the pre-replicative complex	LMO2 gene body methylated status was identified in IGVH-mutated samples	[26, 60–62]
RARb2	Angiogenesis and erythropoiesis gene expression (transcription), Assembly of the prereplicative complex	Hypermethylation of RARbCpG islands was identified in 29.7% of the analyzed samples in a group of patients while in another series, it was found in 3.1% of the samples studied	[32, 51, 63]
CAT	Gene expression (transcription) innate immune system oxidative stress	A distal CpG island in the promoter region remained methylated both in normal B cells and CLL cells, while variable methylation levels were recorded in the proximal CpG island only in CLL cells exposure of CLL cells to a demethylating agent led to increased catalase mRNA levels	[64]
ZNF540	Gene expression (transcription) MAPK pathway	Methylated status of its gene body was reported in IGVH-unmutated cases	[26, 65]

CLL: chronic lymphocytic leukemia, Ref.: reference, ZNF540: human zinc finger protein 540, LMO2: LIM-only protein 2, RARb2: retinoic acid receptor B2, CAT: catalase, CTLA4: cytotoxic T-lymphocyte associated protein 4, MAPK: mitogen-activated protein kinase, IGVH: immunoglobulin variable heavy chain gene.

**Table 4:** RNA polymerase I promoter opening.

Gene names	Related pathway	Findings in CLL	Ref.
MAFB	RNA polymerase I promoter opening, nervous system development regulation of hematopoiesis	Differentially methylated regions of the MAFB gene have been associated with survival features in CLL cohorts where the hypermethylated status was linked to inferior post-treatment survival	[66]
HOXA4	RNA polymerase I promoter opening	Hypermethylated promoter region was associated with the IGVH-unmutated status and inferior clinical outcome, strong predictor of time to first treatment, independent of the IGVH mutational and CD38 expression status	[67, 68]
TET1	RNA polymerase I promoter opening, gene expression (transcription)	TET1 gene body was variously methylated, 5-aza 2′-deoxycytidine exposure to CLL cells led to decreased occupancy of EZH2 over the TET1 promoter and conversely to the loss of TET1 expression, increased expression of specific intronic transcripts associated with decreased TET1 promoter activity	[69]

CLL: chronic lymphocytic leukemia, Ref.: reference, MAFB: V-maf musculoaponeurotic fibrosarcoma oncogene homolog B, HOXA4: homeobox A4, TET1: tet methylcytosine dioxygenase 1, CD38: cluster of differentiation 38, EZH2: enhancer of Zeste 2 polycomb repressive complex 2 subunit, IGVH: immunoglobulin variable heavy chain gene.

**Table 5:** Programmed cell death/cell apoptosis and p53 signaling.

Gene name	Related pathway	Findings in CLL	Ref.
ITGA4	Apoptotic pathways and integrin pathway	Protein levels are related to more aggressive course and shorter time to treatment, protein expression is regulated at the mRNA level and in a methylation-regulated manner, hypermethylation at specific ITGA4 CpG sites was shown to be a common phenomenon in the del13q14+ samples, and the methylation status of ITGA4 at CpG site 1 was demonstrated to be have a prognostic role	[70]
S100A14	Modulates TP53 protein levels MAPK-Erk NF-kB	Unmethylated gene body status was found in IGVH-unmutated samples	[26, 71, 72]
TP53I3	Gene expression (transcription), TP53-mediated transcription of cell death genes	Gene methylated gene body status was reported in IGVH-mutated cases, patients showed the unmethylated TP53 gene promoter status, and TP53 promoter methylation significantly correlated to reduced platelet counts and advanced stage at diagnosis	[26, 73–76]
p73	TP53-mediated transcription of cell death genes; gene expression (transcription)	Protein expression correlated positively with higher risk of CLL stages; regarding p73 hypermethylation, there are conflicting results from different studies showing that pathways other than isolated regulation of p73 activity are responsible for CLL pathogenesis	[32, 77–79]
TP63	TP53-mediated transcription of cell death genes; gene expression (transcription)	TP63 gene was primarily hypomethylated in the promoter region and overexpressed in a subset of IGVH-unmutated samples with the highest risk for Richter's transformation. BCR stimulation in that group of CLL cases led to protein induction and increased cell survival	[80]
TIMP-2/3	GPCR signaling	TIMP-2 and TIMP-3 generally lack hypermethylation	[32, 81, 82]
NGFR	Antiproliferative signals transmission, p75 NTR receptor-mediated signaling	NGFR gene body was reported to be unmethylated in IGVH-mutated cases	[26, 83]
ITGB2	Apoptotic pathways integrin pathway	The grade of methylation was negatively associated with CD18 surface expression; high grade of ITGB2 promoter methylation was found in CLL samples with low CD18 expression, whereas high CD18 expressing CLL cells; in the trisomy 12 subgroup, they were mainly unmethylated at the same region; when proliferating and nonproliferating cells were compared, the ITGB2 promoter methylation was similar among these groups	[84]
OSM	MIF-mediated glucocorticoid regulation, Erk signaling	In a study where cells from CLL, Richter's transformed CLL, and normal B cells were analyzed, OSM displayed significantly higher promoter methylation levels in Richter's syndrome compared to the other groups	[85]
MET	Apoptotic pathways, GPCR signaling	MET expression was reduced in patients with hypermethylated promoter regions and hypomethylated body regions	[37, 86]
TWIST2	Gene expression (transcription), regulation of activated PAK-2 negative regulator of p53	Promoter methylation was reported in IGVH-mutated cases	[87]

CLL: chronic lymphocytic leukemia, Ref.: reference, NGFR: nerve growth factor receptor, S100A14: S100 calcium binding protein A14, TP53I3: tumor protein P53 inducible protein 3, p73: a, TP63: tumor protein 63, TIMP-2/3: tissue inhibitors of metalloproteinase 2 and 3, ITGA4: integrin subunit alpha 4, ITGB2: integrin subunit beta 2, OSM: oncostatin M, MET: mesenchymal epithelial transition receptor tyrosine kinase, TWIST2: twist family basic helix-loop-helix transcription factor 2, p75: p75 neurotrophin receptor, NTR: neurotrophin receptor, TP53: tumor protein 53, MAPK-Erk: mitogen-activated protein kinase—extracellular signal-regulated kinases, NF-kB: nuclear factor kappa-beta, GPCR: G protein-coupled receptor, PAK-2: P21 activated kinase 2, BCR: B cell receptor gene, del: deletion, CD18: cluster of differentiation 18, IGVH: immunoglobulin variable heavy chain gene.

**Table 6:** TGF-*β* pathway.

Gene name	Related pathway	Findings in CLL	Ref.
DCLK2 and TNFRSF1B	TGF-*β* pathway, TNF superfamily-associated functions	Locus-specific hypomethylation of retrotransposons proximal to DCLK2 and TNFRSF1B was detected and higher levels of DCLK2 and TNFRSF1B expression were associated with inferior survival	[88]
BCL2	MIF-mediated glucocorticoid regulation, TGF-*β* pathway	Methylated BCL2 gene body has been reported in IGVH-mutated patients	[26, 89]
IL17RC	NF-kB MAPK pathway	Unmethylated gene body status was identified in IGVH-unmutated patients	[26, 90]
WT1	Transcription suppressor of multiple proteins including M-CSF, TGF-*β*, and RAR-a	WT1 expression was absent suggesting that WT1 expression was lacking in more mature cell lines; other studies revealed that WT1 promoter was methylated to a large extent of CLL patients; gene promoter regions in normal B cells were completely unmethylated	[51, 91, 92]
FMOD	Keratan sulfate biosynthesis, TGF-*β* pathway, hematopoietic stem cells and lineage-specific markers, TCR signaling	FMOD was highly expressed whereas both hypomethylated gene promoter and gene body were identified	[37, 93, 94]

CLL: chronic lymphocytic leukemia, Ref.: reference, TGF-*β*: transforming growth factor-*β*, DCLK2: doublecortin-like kinase 2, TNFRSF1B: tumor necrosis factor receptor superfamily member 1B, BCL2: B cell lymphoma 2, IL17RC: interleukin 17 receptor C, WT1: Wilms tumor 1 protein, FMOD: fibromodulin, TNF: tumor necrosis factor, MIF: macrophage migration inhibitory factor, NF-kB: nuclear factor kappa-beta, MAPK: mitogen-activated protein kinase, M-CSF: macrophage colony-stimulating factor, RAR-a: retinoic acid receptor alpha, TCR: T-cell receptor, IGVH: immunoglobulin variable heavy chain gene.

**Table 7:** WNT and Hedgehog pathways.

Gene name	Related pathway	Findings in CLL	Ref.
WNT5A	Regulation of activated PAK-2, signaling by WNT developmental pathways during embryogenesis	Its expression differs in CLL patients with worse prognosis in the IGHV-mutated subgroup; methylation levels of all the CpG sites in the WNT5A promoter were lowest in the group of the intermediate genome; methylation profile in the memory-like and intermediate genome methylation profile groups were undetectable; WNT5A expression through its promoter methylation correlated with longer treatment-free survival	[95]
WISP3	Signaling by WNT	Gene promoter was preferentially methylated in IGVH-mutated CLL	[14, 96]
LEF1	Regulation of activated PAK-2 signaling by WNT GSK3 signaling	Methylated promoter status correlated with good prognostic features	[14, 97]
PAX9	WNT/Hedgehog/NOTCH signaling	Higher mRNA expression was detected in IGVH-unmutated cases; high expression correlated with higher risk of treatment initiation, shorter time to first treatment, and was predictive of inferior overall survival	[98]
WIF1, DKK3, APC, SFRP1, SFRP2, SFRP4, and SFRP5	Inhibitors of the WNT pathway	Over a half of CLL cases showed aberrant gene body methylation of at least one gene	[99–101]
E-cad	Loss of function contributes to cancer proliferation, invasion, and/or metastasis, signaling by WNT, and regulation of CDH11	Relative to normal B cells, E-cad was minimally or even absently expressed in globally hypermethylated samples; E-cad hypermethylation was always associated with a simultaneous presence of at least one SFRP gene body hypermethylation	[32, 102, 103]
PTCH1	Endochondral ossification signaling by Hedgehog	PTCH1 promoter was reported to be methylated in 46% of the CLL samples examined compared to no case in samples from healthy individuals	[104]

CLL: chronic lymphocytic leukemia, Ref.: reference, WNT: wingless-related integration site, WNT5A: WNT family member 5A, WISP3: WNT inducible signaling pathway protein 3, LEF1: lymphoid enhancer-binding factor 1, PAX9: paired box gene 9, WIF1: WNT inhibitory factor 1, DKK3: Dickkopf WNT signaling pathway inhibitor 3, APC: adenomatous polyposis coli gene, SFRP1–5: secreted frizzled-related proteins 1–5, E-cad: cadherin-1, PTCH1: protein patched homolog 1, PAK-2: p21 activated kinase, GSK3: glycogen synthase kinase-3, CDH11: cadherin 11, IGVH: immunoglobulin variable heavy chain gene.

**Table 8:** NFAT and TCR signaling.

Gene name	Related pathway	Findings in CLL	Ref.
CD5	Hematopoietic stem cells and lineage-specific markers, TCR signaling	CD5 has been reported to be highly expressed in CLL cells and it is demonstrated to be hypomethylated in promoter and body regions	[37, 94]
NFATC1	Regulation of activated PAK-2, activation of cAMP-dependent PKA downstream regulator of the BCR signaling pathway	Through genome-wide DNA methylation, NFATC1 was reported to be globally hypomethylated and, therefore, upregulated NFATC1 promoter hypomethylation correlated inversely with RNA levels and was associated with Binet disease staging and thymidine kinase levels	[105]
PRF1	Enhancer of MAPK pathway, Granzyme pathway, and TCR signaling	Methylated gene body was reported in IGVH-mutated cases	[26, 106]
BCL10	Activation of the NF-kB pathway, regulation of activated PAK-2 TCR signaling	Unmethylated BCL gene body has been reported in IGVH-mutated patients	[26, 107]

CLL: chronic lymphocytic leukemia, Ref.: reference, NFAT: nuclear factor of activated T-cells, TCR: T-cell receptor, CD5: cluster of differentiation, NFATC1: nuclear factor of activated T-cells 1, PRF1: perforin 1, BCL: B-cell lymphoma, PAK-2: p21 activated kinase, PKA: protein kinase A, BCR: B cell receptor, MAPK: mitogen-activated protein kinase, NF-kB: nuclear factor kappa-beta, IGVH: immunoglobulin variable heavy chain gene.

**Table 9:** NF-kB pathway.

Gene name	Related pathway	Findings in CLL	Ref.
CARD15	Activation of the NF-kB pathway, MyD88-dependent cascade, MAPK activation	Gene body methylation was reported in IGVH-mutated samples	[26, 108]
LOC340061	Activation of the NF-kB pathway, cytosolic sensors of pathogen-associated DNA	Methylated gene body status was reported in IGVH-mutated cases	[26, 109]
UNC5CL	Inhibition of NF-kB-dependent transcription and Netrin signaling	Methylated gene body status was identified in some IGVH-mutated patients	[26, 110]
LDOC1	Regulation of the NF-kB pathway and TNF-*α*-mediated pathway to apoptosis	Unmethylated gene body status was found in IGVH-unmutated cases	[26, 111, 112]
URP2	Inhibition of NF-kB pathway, cell apoptosis response to elevated platelet cytosolic Ca^2+^leukocyte-intrinsic Hippo pathway	Methylated URP2 gene body correlated with IGVH-mutated cases	[26, 113]

CLL: chronic lymphocytic leukemia, Ref.: reference, NF-kB: nuclear factor kappa-beta, CARD15: caspase recruitment domain-containing protein 15, LOC340061: early growth response 3, UNC5CL: unc-5 family C-terminal like, LDOC1: leucine zipper downregulated in cancer 1, URP2: urotensin II-related peptide, MyD88: myeloid differentiation primary response protein 88, MAPK: mitogen-activated protein kinase, TNF-*α*: tumor necrosis factor alpha, IGVH: immunoglobulin variable heavy chain gene.

**Table 10:** MAPK/Erk pathway.

Gene name	Related pathway	Findings in CLL	Ref.
ZAP70	NFAT, TCR signaling, Rho GTPases signaling, NF-kB, Erk signaling	Negative prognostic marker in CLL methylation of a CpG site downstream of transcriptional start site correlated with IGVH-mutated and favorable prognosis	[114–118]
ANGPT2	MIF-mediated glucocorticoid regulation, TGF-*β* pathway, Erk, PI3K-Akt signaling	Higher methylation levels of gene promoter in the IGVH-mutated cases, lower degree of methylation associated with particularly poor prognosis, methylation of its promoter leads to gene silencing	[14, 119–121]
ROR1	GPCR signaling, Erk, signaling by WNT	ROR1 expression was upregulated; hypomethylation in the promoter as well as in the body region was demonstrated	[37, 122]
DAPK1	Programmed cell death dimerization of procaspase-8, MAPK-Erk	Promoter hypermethylation associated with the downregulation of DAPK1 expression	[123]
ADAM12	Cell-to-cell and cell-to-matrix interactions, fertilization, muscle tissue development, and neurogenesis EGFR, MAPK-Erk pathway	48% of the samples were partially methylated and 52% were reported to be unmethylated compared to 20% and 80.6%, respectively, in healthy individuals, an association statistically significant	[124]
AIRE	Regulated by the MAPK pathway	Methylated gene body was observed in IGVH-mutated cells	[26, 125]
RASGRP3	RAF/MAPK cascade regulation of activated PAK-2	Methylated gene body correlated with the IGVH-mutated status	[26, 126–128]
PPP1R3A	Beta-adrenergic signaling	Methylated gene body was observed in IGVH-mutated cases	[26, 129]

CLL: chronic lymphocytic leukemia, Ref.: reference, ZAP70: zeta chain of T-cell receptor-associated protein kinase 70, ANGPT2: angiopoietin 2, ROR1: receptor tyrosine kinase-like orphan receptor 1, DAPK1: death-associated protein kinase 1, ADAM12: disintegrin and metalloproteinase domain-containing protein 12, AIRE: autoimmune regulator, RASGRP3: rat sarcoma guanyl releasing protein 3, PPP1R3A: protein phosphatase 1 regulatory subunit 3A, NFAT: nuclear factor of activated T cells, TCR: T-cell receptor, NF-kB: nuclear factor kappa-beta, MAPK: mitogen-activated protein kinase, Erk: extracellular signal-regulated kinase, MIF: macrophage migration inhibitory factor, TGF-*β*: transforming growth factor beta, PI3K-Akt: phosphatidylinositol-3-kinase—protein kinase B, GPCR: G protein-coupled receptor, WNT: wingless-related integration site, RAF: rapidly accelerated fibrosarcoma, EGFR: epidermal growth factor receptor, PAK-2: p21 activated kinase 2, IGVH: immunoglobulin variable heavy chain gene.

**Table 11:** NOTCH signaling.

Gene name	Related pathway	Findings in CLL	Ref.
CREBBP	Regulation of activated PAK-2, NOTCH	Genome-wide methylation profiling identified CREBBP gene body to be differentially methylated	[130]
HDAC4/HDAC9	Notch signaling gene expression (transcription)	Gene promoter was differentially methylated between prognostic CLL subgroups	[14, 131]
RASF10, RASF6, and KIBRA	Notch signaling regulator of the Hippo/SWH pathway	RASF10 gene promoter was frequently methylated, followed by RASF6 KIBRA gene promoter was recurrently methylated and associated with the IGVH-unmutated status and CD38 expression	[132]
FABP7	PKC, MAPK-Erk, triglyceride metabolism, notch signaling	Methylated gene body was observed in IGVH-mutated cases	[26, 133]
NOTCH1	Notch signaling regulation of activated PAK-2	Gene promoter methylation was associated with aggressive clinical types such as Richter's transformation and chemorefractoriness independent predictor of poor prognosis	[14, 134]

CLL: chronic lymphocytic leukemia, Ref.: reference, NOTCH1: neurogenic locus, notch homolog protein 1, HDAC4/HDAC9: histone deacetylases 4 and 9, RASF6/RASF10: rat sarcoma-associated domain family member 6 and 10, KIBRA: kidney and brain expressed protein, FABP7: fatty acid-binding protein 7, CREBBP: cAMP response element-binding protein binding protein, PAK-2: p21 activated kinase 2, SWH: Salvador-Warts-Hippo, PKC: protein kinase C, MAPK-Erk: mitogen-activated protein kinase-extracellular signal-regulated kinase, CD38: cluster of differentiation 38, IGVH: immunoglobulin variable heavy chain gene.

**Table 12:** JAK-STAT pathway.

Gene name	Related pathway	Findings in CLL	Ref.
SOCS-1	Negative regulator of type I and type II interferon and other cytokines, including IL2, IL4, IL6, and leukemia inhibitory factor; Class I MHC-mediated antigen processing and presentation; inhibitor of the JAK-STAT pathway	SOCS-1 gene body was not hypermethylated in any case	[135]
IL19	MIF-mediated glucocorticoid regulation, TGF-*β* pathway, and JAK-STAT pathway	Methylated gene body was reported in IGVH-mutated CLL cells	[26, 136]
IFNB1	Interferons-mediated signaling pathway DDX58/IFIH1-mediated induction of interferon-alpha/beta, JAK-STAT pathway	Methylated gene body was identified in IGVH-mutated samples	[26, 137]
DUSP22	Inhibitor of MAPK and STAT pathways	Silencing of DUSP22 expression in knocked-out cells resulted in increased STAT3 activity. Targeted bisulfite sequencing and methylation-specific PCR detected that the methylation levels of the DUSP22 promoter were significantly lower in DUSP22 knocked-out cells after treatment with demethylating agents, DUSP22 promoter methylation decreased and subsequently, DUSP22 mRNA levels were increased	[138]

CLL: chronic lymphocytic leukemia, Ref.: reference, JAK: Janus kinase, STAT: signal transducer and activator of transcription, IFNB1: interferon beta 1, IL: interleukin, SOCS-1: suppressor of cytokine signaling 1, DUSP22: dual specificity phosphatase 22, DDX58/IFIH1: DExD/H-box helicase 58/interferon induced with helicase C domain 1, MIF: macrophage migration inhibitory factor, TGF-*β*: transforming growth factor-beta, MHC: major histocompatibility complex, MAPK: mitogen-activated protein kinase, IGVH: immunoglobulin variable heavy chain gene.

**Table 13:** PI3K-Akt signaling.

Gene names	Related pathway	Findings in CLL	Ref.
TCL1A	PI3K-Akt signaling AP1 pathway NF-kB ATM	TCL1A promoter was reported to be hypomethylated in CLL cells and correlated with significant TCL1A transcription enhancement	[139]
PHLPP1	PIP3 signaling PI3K-Akt signaling	Low PHLPP1 expression is reported parallel with its mRNA levels; further analysis detected that the end region of exon 1 may be important in the regulation of PHLPP1 expression although low methylation was observed in the promoter region; compared with normal B cells, the CLL cells with absent or low PHLPP1 expression displayed significantly higher CpG methylation levels and more methylated CpG sites compared than normal B cells and PHLPP1-expressing CLL cells; methylation inhibition led to moderate regulation of PHLPP1 expression	[140]

CLL: chronic lymphocytic leukemia, Ref.: reference, TCL1A: T-cell leukemia/lymphoma 1 oncogene, PHLPP1: PH domain and leucine rich repeat protein phosphatase 1, PI3K-Akt: phosphatidylinositol-3 kinase—protein kinase B, AP1: activator protein 1, NF-kB: nuclear factor kappa-beta, ATM: ataxia-telangectasia mutated, PIP3: phosphatidylinositol (3,4,5)-trisphosphate.

**Table 14:** Rho GTPases/Rhodopsin-like receptors.

Gene names	Related pathway	Findings in CLL	Ref.
ET-1	GPCR signaling, class A/1 (Rhodopsin-like receptors), NF-kB	ET-1 is involved in survival, drug resistance, and growth signaling of leukemic cells; basal expression levels of ET-1 are affected when high methylation levels in a region of ET-1 first intron are detected	[141]
PLD1	Cell survival and protection from apoptosis. Glycerophospholipid biosynthesis signaling by Rho GTPases	Unmethylated PLD1 gene body was reported in IGVH-mutated cases	[26, 142]
DLC1	Signaling by Rho GTPases	In advanced Rai stage CLL cases, aberrant methylation of DLC1 promoter was identified in 89.7% of the samples examined	[36]
S1PR4	Involved in cell migration, GPCR signaling, class A/1 (Rhodopsin-like receptors)	In a study where cells from CLL, Richter's transformed CLL and normal B cells were analyzed, and S1PR4 displayed significantly higher promoter methylation levels in Richter's syndrome compared to the other groups	[85]
GHSR	Class A/1 (Rhodopsin-like receptors), GPCR signaling	Remarkable hypermethylation at the promoter region and first exon of the gene was detected; abnormal methylation was able to distinguish with high sensitivity and specificity malignant from normal cells; GHSR hypermethylation was reported to be identified even in early disease stages	[143]

CLL: chronic lymphocytic leukemia, Ref.: reference, PLD1: phospholipase D1, DLC1: deleted in liver cancer 1, S1PR4: sphingosine 1-phosphate receptor 4, ET-1: endothelin 1, GHSR: growth hormone secretagogue receptor type 1, GPCR: G protein-coupled receptor, NF-kB: nuclear factor kappa-beta, IGVH: immunoglobulin variable heavy chain gene.

**Table 15:** Class I MHC-mediated antigen processing.

Gene names	Related pathway	Findings in CLL	Ref.
VHL	Class I MHC-mediated antigen processing and presentation	Methylated promoter status was reported in IGVH-unmutated CLL	[14, 144]
UBE2R2	Class I MHC-mediated antigen metabolism of proteins	Methylation levels in specific CpG sites independently predicted time to treatment with some of them being located in the gene body	[29]

CLL: chronic lymphocytic leukemia, Ref.: reference, MHC: major histocompatibility complex, VHL: von Hippel–Lindau, UBE2R2: ubiquitin conjugating enzyme E2 R2, IGVH: immunoglobulin variable heavy chain gene.

**Table 16:** ESR-mediated signaling.

Gene names	Related pathway	Findings in CLL	Ref.
MYB	Gene expression (transcription) ESR-mediated signaling, PI3K-Akt signaling, RNA polymerase I promoter opening	Methylated promoter status in IGVH-mutated cases	[14, 145]
CHD1	ESR-mediated signaling chromatin regulation	Hypermethylated gene promoter reported in CLL samples	[102, 103]
ESR1	PI5P, PP2A, and IER3 pathways, PI3K/Akt signaling, ESR-mediated signaling	ESR1 expression was amplified in 15% of the samples examined with copy number losses frequency ranging between 1 and 10%; ESR1 promoter region was methylated in one out of ten CLL samples controlled; in normal B cells, gene promoter was completely unmethylated	[51, 146–148]

CLL: chronic lymphocytic leukemia, Ref.: reference, ESR1: estrogen receptor 1, MYB: myeloblastosis, CHD1: chromodomain helicase-DNA-binding protein 1, PI3K-Akt: phosphatidylinositol-3 kinase—protein kinase B, PI5P: phosphatidylinositol 5 phosphate, PP2A: protein phosphatase 2A, IER3: immediate early response 3, IGVH: immunoglobulin variable heavy chain gene.

**Table 17:** Angiogenesis/erythropoiesis.

Gene names	Related pathway	Findings in CLL	Ref.
SERPINB5	TAp63 isoforms transcription angiogenesis	Methylated gene body was described in IGVH-unmutated cases	[26, 149]
THBS1	Involved in platelet aggregation, angiogenesis, and tumorigenesis gene expression (transcription). Reactive oxygen species signaling. Nitrous oxide signaling	THBS1 promoter methylation was detected in 50% of the samples while in normal samples, gene promoters were mainly unmethylated	[51, 150, 151]
TJP1	Immune cell transmigration VCAM-1/CD106 signaling gene expression (transcription); MAGuK tumor suppressor pathway	TJP1expression was reduced in patients with hypermethylated promoter regions and hypomethylated body regions	[37, 152]

CLL: chronic lymphocytic leukemia, Ref.: reference, SERPINB5: serine protease inhibitor B5, THBS1: thrombospondin-1, TJP1: tight junction protein 1, Tap63: tumor protein P63, VCAM-1: vascular cell adhesion protein 1, CD106: cluster of differentiation 106, MAGuK: membrane-associated guanylate kinases, IGVH: immunoglobulin variable heavy chain gene.

**Table 18:** SLIT/ROBO signaling and nervous system development.

Gene names	Related pathway	Findings in CLL	Ref.
SLIT2	Nervous system development; regulation of SLIT/ROBO and VEGF signaling pathways	Frequent promoter methylation was reported in high-risk CLL cases	[98, 153, 154]
ROBO 1	Nervous system development, SLIT/ROBO signaling pathways	The expression of ROBO1 was significantly lower compared to healthy control samples; when DNA sequencing of the promoter region was performed, in most CLL samples, a higher level of methylation than the healthy individuals was identified	[155]
ID4	Signaling by NTRKs nuclear events (kinase and transcription factor activation)	Shortened patient survival is associated with high levels of promoter; methylation ID4 promoter methylation is generally methylated in varying levels in CLL cells; ID4 mRNA and protein expression are both silenced in CLL cells, a phenomenon independent of ID4 promoter methylation status	[156–158]
PTPRO	Signaling by the NTRKs PAK pathway	PTPRO gene promoter was methylated and expression was suppressed compared to normal B cells, with overall expression of PTPRO being lower in CLL lymphocytes than in normal samples	[51, 159–161]

CLL: chronic lymphocytic leukemia, Ref.: reference, SLIT2: slit glycoprotein 2, ROBO1: roundabout receptor 1, ID4: inhibitor of differentiation 4, PTPRO: protein tyrosine phosphatase type O, SLIT/ROBO: slit glycoprotein/roundabout receptor, VEGF: vascular endothelial growth factor, NTRK: neurotrophic tyrosine receptor kinase, PAK: p21 activated kinase, IGVH: immunoglobulin variable heavy chain gene.

**Table 19:** Drug uptake mechanisms and sensitivity.

Gene names	Related pathway	Findings in CLL	Ref.
SLC22A18	Drug sensitivity cellular metabolism and growth	Unmethylated gene body status was reported in IGVH-unmutated samples	[26, 162]
SLCO3A1	Transport of inorganic cations/anions and amino acids/oligopeptides and transport of vitamins, nucleosides, and related molecules, drug uptake mechanisms	Differentially methylated regions of the SLCO3A gene have been associated with survival features in CLL cohorts where the hypermethylated status was linked to inferior post-treatment survival	[66]

CLL: chronic lymphocytic leukemia, Ref.: reference, SLC22A18: solute carrier family 22, member 18, SLCO3A1: solute carrier organic anion transporter family member 3A1, IGVH: immunoglobulin variable heavy chain gene.

**Table 20:** Various other genes investigated in CLL.

Gene names	Related pathway	Findings in CLL	Ref.
LPL	Statin inhibition of cholesterol production; familial hyperlipidemia type 1 pathways	Prognostic marker in CLL gene promoter methylation was associated with IGVH-mutated cases and longer time to treatment	[14, 163, 164]
ABI3	Possible interaction with p21activated kinase inhibits ectopic metastasis of tumor cells	Gene promoter and gene body were frequently methylated in IGVH-unmutated CLL	[14, 26, 165]
CRY1	Melatonin metabolism	Higher mRNA expression was detected in the IGVH-unmutated subgroup; treatment initiation was significantly more frequent among patients with high expression of CRY1; high expression was significantly associated with shorter intervals to first treatment	[98, 153, 154]
SCGB2A1	Androgen receptor signaling pathway	Methylated gene body status was reported in IGVH-unmutated cases	[26, 166]
GATA5	Cooperative activation of the intestinal lactase-phlorizin hydrolase promoter response to elevated platelet cytosolic Ca^2+^	GATA 5 promoter hypermethylation was detected in 35.1% of the cases	[51, 167]
DNTT	MYC transcriptional repression DNA-PK pathway	Methylated DNTT gene body was described in IGVH-mutated samples	[26, 168, 169]
SHANK1	Protein-protein interactions at synapses; transmission across chemical synapses	A specific CpG region of the SHANK1 body (hg19) was reported to be hypermethylated when compared to control sample methylation in the same. CpG was detectable in blood samples collected years before CLL diagnosis	[170]
SYN2	Neurotransmitter release cycle; transmission across chemical synapses	SYN2 expression was demonstrated to be reduced in patients with hypermethylated promoter regions and hypomethylated body regions	[37, 171]

CLL: chronic lymphocytic leukemia, Ref.: reference, LPL: lipoprotein lipase, ABI3: Abelson interactor family member 3, CRY1: cryptochrome 1, SCGB2A1: secretoglobin family 2A member 1, GATA5: GATA-binding protein 5, DNTT: DNA nucleotidylexotransferase, SHANK1: SH3 and multiple ankyrin repeat domain 1, SYN2: synapsin II, MYC: MYC proto-oncogene, DNA-PK: DNA-dependent protein kinase, IGVH: immunoglobulin variable heavy chain gene.

**Table 21:** Various other genes were investigated in CLL (cont.).

Gene names	Related pathway	Findings in CLL	Ref.
GAB2	Insulin receptor signaling and FLT3 signaling	Genome-wide methylation profiling identified the GAB2 gene body to be differentially methylated	[130]
OSBPL1A	Synthesis of bile acids and bile salts metabolism	OSBPL1A expression was reduced in patients with hypermethylated promoter regions and hypomethylated body regions	[37, 172]
IL1B	MIF-mediated glucocorticoid regulation	Methylation levels in specific CpG sites independently predicted time to treatment with some of them being located in the gene body	[29]
UBC	Regulation of activated PAK-2, MyD88-dependent cascade	Genome-wide methylation profiling identified UBC gene body to be differentially methylated	[130]
IGSF4/TSLC1	Involved in cell adhesion and metastatic potential, MAGuK tumor suppressor pathway and regulation of CDH11	The frequency of gene promoter hypermethylation reached 62.2% of the samples studied methylated status of IGSF4 promoter has been reported in IGVH-unmutated cells	[51, 173–175]
GPX3	Glutathione conjugation cellular responses to stimuli	Methylated gene body status was documented in IGVH-unmutated cases	[26, 176]
FHL2	PPARA activates gene expression repressive role in beta-chain gene expression	Methylated FHL2 gene body was reported in IGVH-unmutated cases	[26, 177, 178]

CLL: chronic lymphocytic leukemia, Ref.: reference, GAB2: growth factor receptor-bounding protein 2-associated binder 2, OSBPL1A: oxysterol-binding protein like 1A, IL1B: interleukin1B, UBC: induction of ubiquitin C, IGSF4/TSLC1: immunoglobulin superfamily member 4/tumor suppressor in lung cancer, GPX3: plasma glutathione peroxidase, FHL2: 4-and-a-half LIM domain protein 2, FLT3: FMS‐like tyrosine kinase 3, MIF: macrophage migration inhibitory factor, PAK-2: p21 activated kinase 2, MyD88: myeloid differentiation primary response protein 88, MAGuK: membrane-associated guanylate kinase, CDH11: cadherin 11, PPARA: peroxisome proliferator-activated receptor alpha, IGVH: immunoglobulin variable heavy chain gene.

**Table 22:** Various other genes were investigated in CLL (cont.).

Gene names	Related pathway	Findings in CLL	Ref.
REPS1	Vesicle-mediated transport and clathrin-mediated endocytosis	Methylation levels in specific CpG sites independently predicted time to treatment with some of them being located in the gene body	[29]
ATP9B	Ion channel transport and cardiac conduction	Methylation levels in specific CpG sites independently predicted time to treatment with some of them being located in the gene body	[29]
GRB2	Downstream signaling of activated FGFR2 and prolactin signaling	Genome-wide methylation profiling identified GRB2 gene body to be differentially methylated	[130]
DLEU7		Analysis did not detect any specific mutations in DLEU7, but DLEU7 expression was absent in CLL cells; methylation of a CpG island in the promoter region of DLEU7 was identified as a possible explanation for the absence of DLEU7 expression, with the promoter reported to be methylated in most of the CLL samples	[179]
ADAMTS17	Metabolism of proteins immune cell transmigration, VCAM-1/CD106 signaling, gene expression (transcription). MAGuK tumor suppressor pathway	ADAMTS17 expression was reduced in patients with hypermethylated promoter regions and hypomethylated body regions	[37, 180]
KCNG2	Integration of energy metabolism potassium channels	KCNG2 expression was reported to be reduced in patients with hypermethylated promoter regions and hypomethylated body regions	[37, 181]
ME3	Respiratory electron transport ATP synthesis TCA cycle	ME3 expression was reported to be reduced in patients with hypermethylated promoter regions and hypomethylated body regions	[37, 182]

CLL: chronic lymphocytic leukemia, Ref.: reference, REPS1: RalA-binding protein-associated Eps domain-containing 1, ATP9B: ATPase phospholipid transporting 9B, GRB2: growth factor receptor-bounding protein 2, DLEU7: deleted in lymphocytic leukemia 7, ADAMTS17: a disintegrin and metalloproteinase with thrombospondin motifs 17, KCNG2: potassium voltage-gated channel modifier subfamily G member 2, ME3: malic enzyme 3, FGFR2: fibroblast growth factor receptor 2, VCAM-1: vascular cell adhesion protein 1, CD106: cluster of differentiation 106, MAGuK: membrane-associated guanylate kinase, ATP: adenosine triphosphate, TCA: tricarboxylic acid.

**Table 23:** Noncoding RNAs.non

Gene names	Related pathway	Findings in CLL	Ref.
CRNDE	Involved in the expression of genes associated to metabolism and in neoplasms biology inversely regulated by insulin and insulin-like growth factors	CRNDE expression was downregulated and the methylation level of CRNDE promoter was higher compared to normal B cells; after exposure to demethylating agents, an increase of CRNDE expression levels was reported	[183]
NCRMS		Methylated gene body status correlated with IGVH-mutated status	[26, 184]
BM742401		Overexpression of BM742401 in CLL led to interruption of cellular proliferation and increased apoptosis; in CLL cell lines, BM742401 promoter was methylated in 57.1% of the samples compared to normal controls where the promoter was unmethylated; methylation of BM742401 negatively associated with its expression and 5-aza-2′-deoxycytidine exposure caused promoter demethylation with consequent activation of BM742401 expression; methylation of BM742401 correlated with higher Rai stage among in the subgroup of CLL patients with standard-risk cytogenetic features	[185]
CLLU1		mRNA expression level predicted time to treatment and survival; methylated gene promoter correlated with decreased expression and IGVH-mutated cases	[14, 186]
AC012065.7	Positive expression relationship with nearby GDF7 gene. GDF7 protein is involved in growth, repair, and embryonic development	Gene promoter methylation levels reported to be inversely correlated to gene expression levels and survival analysis demonstrated that hypomethylated gene promoter status of AC012065.7 was associated with an inferior prognosis	[187]

CLL: chronic lymphocytic leukemia, Ref.: reference, CLLU1: chronic lymphocytic leukemia upregulated 1, NCRMS: noncoding RNA in rhabdomyosarcoma, BM742401: GATA6 antisense RNA 1, CRNDE: colorectal neoplasia differentially expressed, GDF7: growth differentiation factor 7, IGVH: immunoglobulin variable heavy chain gene.

## Data Availability

The datasets used to support the findings of this study are available from the corresponding author on reasonable request.
